# MicroRNA-629-5p promotes osteosarcoma proliferation and migration by targeting caveolin 1

**DOI:** 10.1590/1414-431X202010474

**Published:** 2021-04-19

**Authors:** Chunsheng Gao, Jun Gao, Ge Zeng, Huichao Yan, Junhua Zheng, Weichun Guo

**Affiliations:** 1 Department of Orthopedics, Renmin Hospital of Wuhan University, Wuhan, Hubei, China; 2 Department of Orthopedics, The Third People's Hospital of Hubei Province, Wuhan, Hubei, China; 3 Department I of Orthopedics, Renmin Hospital of Wuhan University, Wuhan, Hubei, China

**Keywords:** Osteosarcoma, miR-629-5p, CAV1, Proliferation, Migration

## Abstract

Osteosarcoma is a highly malignant tumor that occurs in the bone. Previous studies have shown that multiple microRNAs (miRNAs) regulate the development of osteosarcoma. This study aimed to explore the role of *miR-629-5p* and its target gene, *caveolin 1* (*CAV1*), in osteosarcoma development. To analyze the expression of *miR-629-5p* and *CAV1* mRNA in osteosarcoma tissues and cell lines, qRT-PCR analysis was performed. Dual-luciferase reporter experiments were subsequently performed to validate the relationship between *CAV1* and *miR-629-5p*. CCK8 assay was used to measure osteosarcoma cell proliferation, and wound-healing assay was performed to study their migratory phenotype. Our findings revealed that *miR-629-5p* was overexpressed in osteosarcoma tissues and cells, and thereby enhanced cell proliferation and migration. Further, we validated that *miR-629-5p* targets *CAV1* mRNA directly. *CAV1* expression, which was negatively correlated with *miR-629-5p* expression, was found to be downregulated in osteosarcoma tissue samples. Moreover, our data showed that an increase in *CAV1* level led to a decline in osteosarcoma cell proliferation and migration, which could be rescued by *miR-629-5p* upregulation. Overall, our study confirmed that *miR-629-5p* promoted osteosarcoma proliferation and migration by directly inhibiting *CAV1*.

## Introduction

Osteosarcoma is a malignant bone tumor that typically develops in the knees (1). Osteosarcoma is the most common type of bone cancer in children and adolescents under the age of 20, and is a life-threatening cancer (2,3). In recent years, advances in diagnostics and treatments have considerably improved the survival rate and quality of life of patients with osteosarcoma (4). Much progress has also been made in decreasing osteosarcoma symptoms owing to the application of chemotherapy, radiotherapy, and new anti-tumor therapies (5-7). However, the cure rate of high-risk osteosarcoma remains low due to drug resistance and metastasis (1). These continue to be major challenges in the successful treatment of osteosarcoma. Therefore, there is an urgent need for further research to identify new treatment modalities for osteosarcoma.

Mature microRNAs (miRNAs) are formed by splicing of precursors encoded by endogenous genes, and many studies have shown that although miRNAs are not directly involved in translation, they have important biological roles such as in the formation of silencing complexes (8,9). As gene regulators, miRNAs induce mRNA degradation or inhibit translation (10,11). Some studies suggest that miRNAs function as oncogenes or tumor suppressor genes in metastasis and development of human cancers (12-14). A growing body of research suggests that multiple miRNAs are involved in osteosarcoma tumorigenesis (15,16). A previous study found that abnormally reduced *miR-629-5p* expression markedly stimulated the aggressiveness of colorectal cancer (17). In another study, *miR-629-5p* was identified as a regulator of the development and metastasis of hepatocellular carcinoma (18). Moreover, previous studies have shown that high *miR-629* expression predicts poor prognosis and promotes cell proliferation, migration, and invasion in osteosarcoma (19). Nonetheless, the downstream target genes of *miR-629-5p* have not yet been fully investigated.

*Caveolin1* (*CAV1*) constitutes the main structural protein component of caveolae that play a crucial role in various biological processes by interacting with various proteins and non-protein molecules (20,21). Previous studies have shown that inhibition of *CAV1* significantly increased the resistance of ovarian cells to paclitaxel (22). *CAV1* inhibited the proliferation, migration, and invasion of gastric cancer cells (23). In osteosarcoma, *CAV1* was found to be significantly decreased in high-grade osteosarcoma compared to normal controls (24). In another study, high metastatic mouse osteosarcoma FBJ-S1 cells were found to have lower expression of *CAV1* compared to low metastatic FBJ-LL cells (25). Another study reported the negative effect of *CAV1* on osteosarcoma cells by reducing cell proliferation and invasion (26). Taken together, these results indicate that *CAV1* acts as a tumor suppressor in osteosarcoma. Bioinformatics analysis revealed that low expression of *CAV1* was a key regulator of osteosarcoma and may be the target gene of *miR-629-5p*.

As the relationship between *CAV1* and *miR-629-5p* in osteosarcoma has not been examined previously, this study aimed to identify the roles of *miR-629-5p* and its potential target gene, *CAV1*, in osteosarcoma cell phenotypes. It also aimed to determine the underlying molecular mechanisms of osteosarcoma development. The findings of our study may provide further insight into potential molecular targets for osteosarcoma treatments.

## Material and Methods

### Tissue acquisition and cell culture

All osteosarcoma tissue samples used in this study were collected from the Renmin Hospital of the Wuhan University, and the study protocol (approval No. WDRY2015-K419) was approved by the Ethics Committee of the Renmin Hospital of the Wuhan University. The clinical parameters of osteosarcoma patients are shown in Table 1. Osteosarcoma cell lines (HOS, Saos2, U2OS, and SJSA-1) and immortalized osteoblast line (hFOB1.19) were purchased from the American Type Culture Collection (USA). Cells were cultured under 5% CO_2_ at 37°C in Dulbecco's modified eagle medium (cat. No. 11320033; Gibco; Thermo Fisher Scientific Inc., USA) with 10% fetal bovine serum (cat. No. 16140071; Gibco; Thermo Fisher Scientific Inc.) and 100 U/mL streptomycin (cat. No. 85886; Sigma, China).

**Table 1:** Clinical parameters of patients with osteosarcoma in this study.

Clinical parameters	n (%)
Gender	
Male	8 (42.1%)
Female	11 (57.9%)
Age	
≥25	10 (53.6%)
<25	9 (47.4%)
Location	
Distal of femur	9 (47.4%)
Proximal of tibia	8 (42.1%)
Other	2 (10.5%)
Enneking stage	
I	10 (52.6%)
II	6 (31.6%)
III	3 (15.8%)
TNM	
I+II	14 (73.7%)
III+IV	5 (26.3%)
Distant metastasis	
Negative	16 (84.2%)
Positive	3 (15.8%)

### Quantitative reverse transcription-polymerase chain reaction (qRT-PCR)

Total RNA was isolated using TRIzol reagent (cat. No. 15596026; Thermo Fisher Scientific Inc.). Then, the isolated RNAs were reverse transcribed into cDNA. *miR-629-5p* was reverse transcribed using the mirVana qRT-PCR miRNA Detection kit (cat. No. AM1558; Thermo Fisher Scientific Inc.) according to the manufacturer's instructions. *CAV1* was reverse transcribed using SuperScript™ III First-Strand Synthesis SuperMix for qRT-PCR (cat. No. 11752050; Thermo Fisher Scientific Inc.). qRT-PCR was then performed using the StepOnePlus™ Real-Time PCR System (cat. No. 4376600; Thermo Fisher Scientific Inc.). The data were analyzed using the 2^-ΔΔCt^ method, with U6 (NR_004394.1) and β-actin (241bp, NM_001172897.2) serving as internal controls for *miR-629-5p* (miRBase ID: hsa-*miR-629-5p*, MIMAT0004810) and *CAV1* (135 bp, NM_205518.1), respectively. Primer sequences are shown in Table 2.

**Table 2:** Primer sequences used in this study.

Primer	Sequences
miR-629-5p	
Forward	5′-TGGGTTTACGTTGGGAGA-3′
Reverse	5′-GTGCAGGGTCCGAGGTATTC-3′
CAV1	
Forward	5′-CTGTCGGAGCGGGACATCT-3′
Reverse	5′-GCCTTCCAAATGCCGTCAAA-3′
β-actin	
Forward	5′-CACCATTGGCAATGAGCGGTTC-3′
Reverse	5′-AGGTCTTTGCGGATGTCCACGT-3′
U6	
Forward	5′-TGCGGGTGCTCGCTTCGGCAGC-3′
Reverse	5′-CCAGTGCAGGGTCCGAGGT-3′

### Cell transfection

*miR-629-5p* mimic, *miR-629-5p* inhibitor, and *miR-629-5p* negative control were purchased from Guangzhou RiboBio Co., Ltd. (China) (Supplementary Table S1). *CAV1*-overexpression (*CAV1* OE) plasmid was constructed by cloning full length *CAV1* gene into pcDNA3.1 vector (GeneCopoeia Inc., cat. No. T2806; China). Next, HOS and Saos2 cells were transiently transfected with 75 nM *miR-629-5p* mimic, negative control, *miR-629-5p* inhibitor, and 3 μg *CAV1* OE plasmid using Lipofectamine 2000 (cat. No. 11668019; Thermo Fisher Scientific Inc.) at room temperature. After the cells were incubated for 2 days at 37°C, they were analyzed by qRT-PCR.

### CCK-8 assay

Cells (100 μL) suspended in logarithmic growth phase were plated in 96-well plates (2000 cells/well) and cultured at 37°C for the appropriate duration of 12, 24, 48, and 72 h. Then, 10 μL CCK-8 solution was added to each well according to the CCK-8 user manual (cat. No. E606335; Sangon, China). After the cells were incubated with CCK-8 for 2 h, the absorbance was measured at 450 nm.

### Wound-healing assay

Cells (2×10^5^ cells/well) were plated in 6-well plates and cultured until they reached 80-90% confluence. The cell monolayer was scratched in a straight line with a sterile 200 μL pipette tip to simulate a wound, and the scraped off cells were removed. The medium was replaced with serum-free media containing 10 μg/mL of mitomycin-C to stop proliferation. The cells in the blank control (CON) group were not treated with mitomycin-C. Cells were photographed at 0 and 24 h. The migrated distance (i.e., scratch width at 0 h - scratch width at 24 h) from the edge of the scratch was measured using Adobe Illustrator, a software application (USA).

### Bioinformatics analysis

GSE11414 and GSE12865 data series were downloaded from the gene expression omnibus (GEO) database (https://www.ncbi.nlm.nih.gov/gds/?term=). GSE11414 (27) included four osteosarcoma cell samples (two MG63 cell line samples and two U2OS cell line samples) and two normal human osteoblasts samples (two HOB cell line samples). GSE12865 (28) included twelve osteosarcoma tissue samples and two primary normal human osteoblast cell samples, which were obtained from healthy male donors. The two data series were analyzed using the GEO2R algorithm built into the GEO database (https://www.ncbi.nlm.nih.gov/geo/). The differentially expressed genes (DEGs) in GSE11414 were identified using the criteria of adjusted P<0.05 and |logFC|≥1.5, whereas those in GSE12865 were identified using the criteria of adjusted P<0.05 and |logFC|≥2. TargetScan Human 7.2, an online tool (http://www.targetscan.org/vert_72/), was used to predict potential target genes of *miR-629-5p*. Then, the overlapping genes from GSE12865 DEGs, GSE11414 DEGs, and the target genes of *miR-629-5p* by Venny 2.1.0 (https://bioinfogp.cnb.csic.es/tools/venny/) were selected. Finally, the overlapping genes were uploaded to the STRING algorithm (https://string-db.org/) to identify the key gene.

### Luciferase reporter assay

First, full length *CAV1* 3′UTR (wild-type) or its mutant (*CAV1*-MUT, 205-211 sequence) were cloned into psiCHECK-2 vector (GenePharma, China). HOS and Saos2 cells were then co-transfected with *miR-629-5p* mimic and wild-type *CAV1* or *CAV1*-MUT luciferase reporter plasmids. After 48 h of incubation, the culture medium was removed and cells were collected. The collected cells were lysed to generate cell lysates. Luciferase activity was measured using the Pierce™ Renilla-Firefly Luciferase Dual Assay kit (cat. No. 16185; Thermo Fisher Scientific Inc.) according to the manufacturer's guidelines.

### Statistical analysis

All means and standard deviations were calculated based on three independent experiments. One-way analysis of variance (ANOVA) was used for statistical analysis between multiple groups. Mann-Whitney test was used for statistical analysis of RNA expression in tissue samples. P<0.05 was considered statistically significant.

## Results

### Upregulation of *miR-629-5p* in osteosarcoma

**Figure 1:**
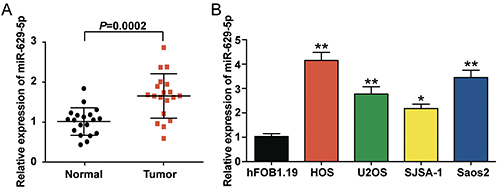
*miR-629-5p* was upregulated in osteosarcoma tissues and cells. **A**, The relative expression level of *miR-629-5p* was analyzed using qRT-PCR in osteosarcoma (n=19) and adjacent healthy tissues (n=19). *miR-629-5p* was upregulated in osteosarcoma tissues. U6 was used as the internal control. **B**, The expression of *miR-629-5p* in osteosarcoma cell lines (HOS, U2OS, SJSA-1, and Saos2) and an immortalized normal osteocyte cell line (hFOB1.19) was determined by qRT-PCR. U6 was used as the internal control. Data are reported as mean±SD. *P<0.05, **P<0.001 compared with hFOB1.19 cells (ANOVA).

Examination of U6 C_T_ values in osteosarcoma tissues and cell lines revealed no significant difference in the C_T_ values between that of osteosarcoma and adjacent normal tissues. Furthermore, we observed that the U6 C_T_ values of HOS, U2OS, SJSA-1, and Saos2 were similar to those of hFOB1.19 (Supplementary Figure S1A and B). These results showed that U6 was stably expressed in osteosarcoma tissues and cell lines. Thus, U6 was used as the internal reference for *miR-629-5p* in subsequent experiments. *miR-629-5p* expression was analyzed in osteosarcoma (n=19) and adjacent normal tissues (n=19) using qRT-PCR. The relative expression level of *miR-629-5p* was significantly higher in osteosarcoma tissues than that in the adjacent healthy tissues (P=0.0002) (Figure 1A). We also observed that osteosarcoma cell lines (HOS, U2OS, SJSA-1, and Saos2) displayed higher expression levels of *miR-629-5p* compared to the immortalized osteoblast cell line hFOB1.19 (Figure 1B). In addition, Saos2 and HOS cell lines showed higher *miR-629-5p* expression levels compared to the other osteosarcoma cell lines (P<0.001). Therefore, Saos2 and HOS cells were selected for subsequent experiments. In summary, *miR-629-5p* was upregulated not only in osteosarcoma tissues but also in cell lines.

### Effect of *miR-629-5p* in osteosarcoma

**Figure 2:**
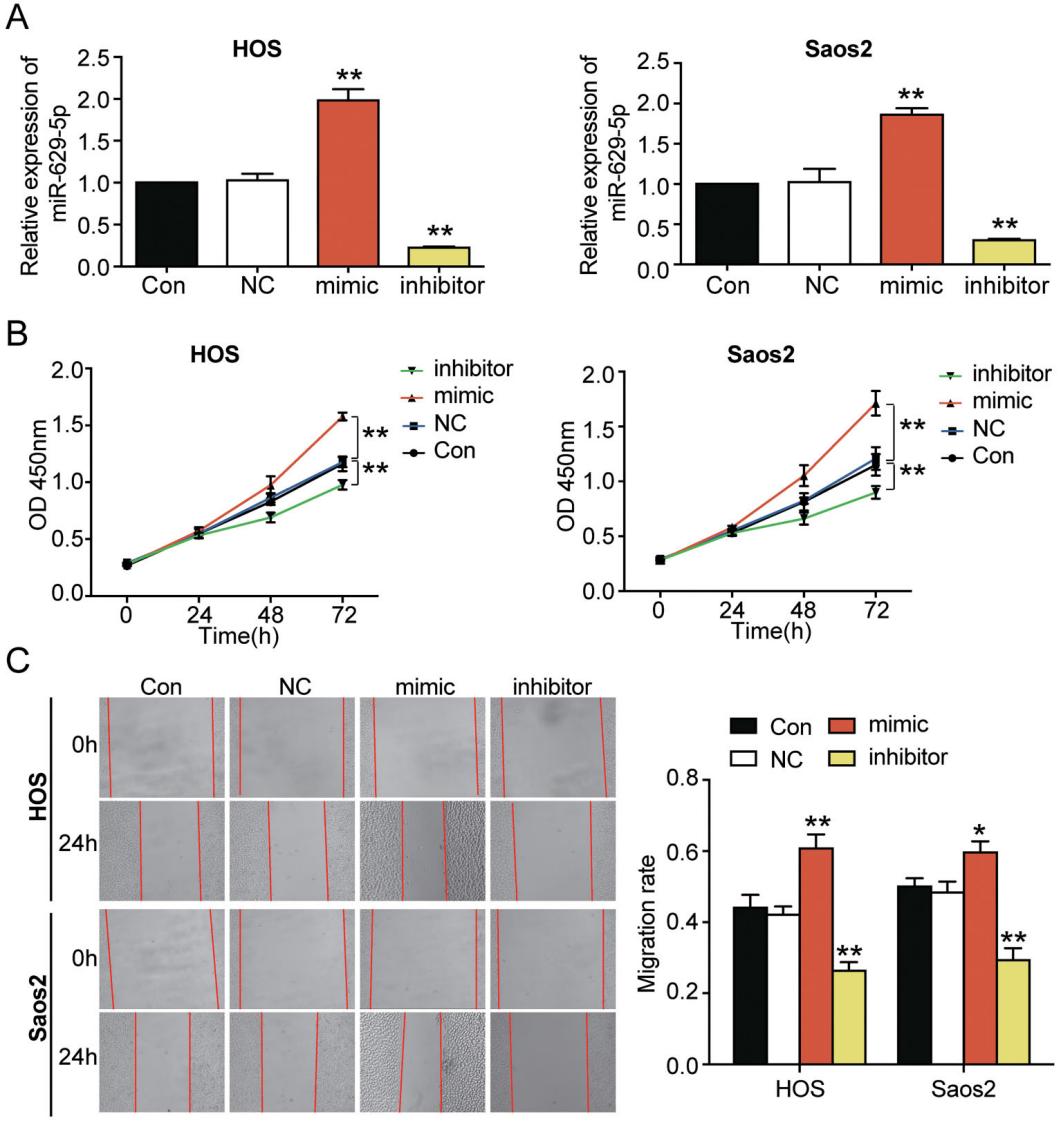
*miR-629-5p* upregulation promoted proliferation and migration of HOS and Saos2 cell lines. **A**, *miR-629-5p* mimic transfection significantly increased *miR-629-5p* expression, while *miR-629-5p* inhibitor significantly reduced it, thus indicating successful cell transfection. **B**, After the *miR-629-5p* mimic or inhibitor was transfected into HOS and Saos2 cells, the proliferation was determined using CCK-8 assay. **C**, The migration phenotypes of the HOS and Saos2 cells transfected with *miR-629-5p* mimic or inhibitor were detected with wound-healing assay. CON: blank control; NC: negative control; Mimic: *miR-629-5p* mimic; Inhibitor: *miR-629-5p* inhibitor. **A**-**C**, The data of the three independent experiments are reported as means±SD. *P<0.05, **P<0.001 compared with control group (ANOVA).

The qRT-PCR analysis revealed that transfection with the *miR-629-5p* mimic markedly increased the expression of *miR-629-5p*, while transfection with the *miR-629-5p* inhibitor reduced *miR-629-5p* expression. These results indicated successful transfection of both cell lines (P<0.001) (Figure 2A). The CCK-8 assay was used to evaluate the rate of proliferation of the transfected cells. CCK-8 results showed that upregulation of *miR-629-5p* significantly enhanced the proliferation of Saos2 and HOS cells, while downregulation of *miR-629-5p* markedly weakened the proliferation rate (P<0.001) (Figure 2B). In addition, *miR-629-5p* upregulation increased cell migration (P<0.05), whereas *miR-629-5p* downregulation inhibited it (P<0.001) (Figure 2C). These results confirmed that *miR-629-5p* significantly increased the malignant phenotypes of osteosarcoma cells.

### *CAV1* was a target of *miR-629-5p*

**Figure 3:**
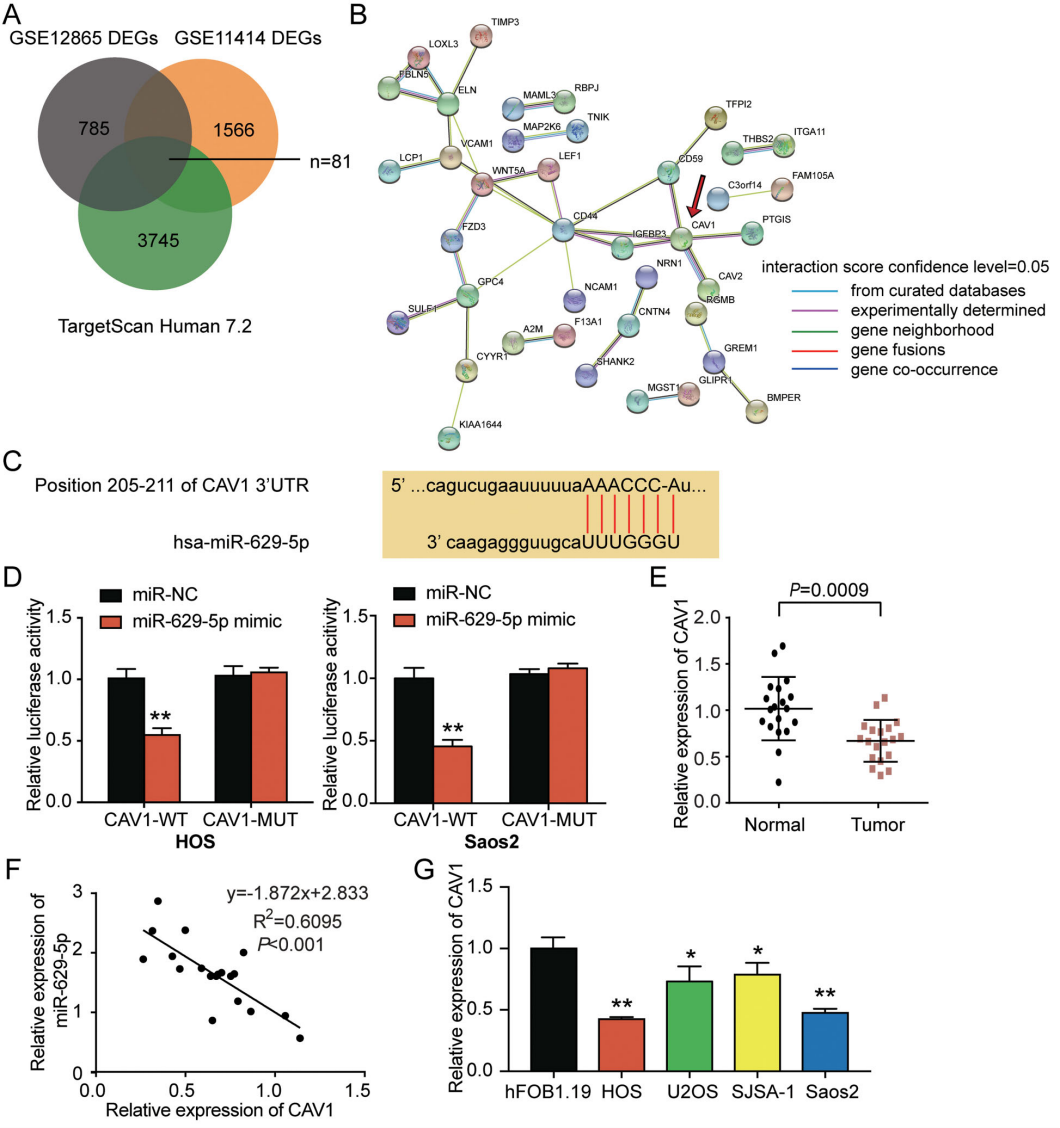
*miR-629-5p* directly targeted *CAV1*. **A**, Intersection between differentially expressed genes (DEGs) from GSE11414 and GSE12865 and the predicted targets of *miR-629-5p* by TargetScan Human 7.2. **B**, STRING interrogation output of the 81 common genes from the last step. **C**, TargetScan Human 7.2 was used to predict the potential binding site between *miR-629-5p* and *CAV1*. **D**, Potential binding between *miR-629-5p* and 3′UTR of *CAV1* was validated by dual-luciferase reporter assay in HOS and Saos2 cell lines. *miR-629-5p* mimic attenuated the fluorescent activity of *CAV1*-WT but did not affect the fluorescent activity of *CAV1*-MUT. **P<0.001 compared with the co-transfection of the *CAV1*-WT group and the NC group. **E**, The expression level of *CAV1* in osteosarcoma tissues and normal tissues was measured by qRT-PCR. **F**, The relationship between *miR-629-5p* and *CAV1* was identified using Pearson correlation analysis. **G**, The expression of *CAV1* in osteosarcoma cell lines and hFOB1.19 cell line. β-actin was used as the internal control. *CAV1*-WT: wild-type *CAV1*; *CAV1*-MUT, *CAV1* mutant; NC: negative control. *P<0.05, **P<0.001 compared with hFOB1.19 cells (ANOVA). **D** and **G**, The data of the three independent experiments are reported as means±SD.

To identify the potential downstream interacting targets of *miR-629-5p* that participate in osteosarcoma pathogenesis, we downloaded two GEO expression datasets (GSE12865 and GSE11414) and analyzed the common differentially expressed genes (DEGs) (Supplementary Figure S2A and B). We found 81 common genes that were significant DEGs in osteosarcoma, including those with potential binding targets of *miR-629-5p* (Figure 3A). We hypothesized that these 81 genes may have significant roles in osteosarcoma progression. Therefore, we used the STRING algorithm to analyze the interaction between the 81 genes. The results showed that *CAV1* had the greatest number of, and the most evident interactions with, other genes in the network (Figure 3B). Therefore, we selected *CAV1* for further analysis and retrieved the binding sequence between *miR-629-5p* and *CAV1* from TargetScan Human 7.2 (Figure 3C). To further ascertain the relationship between *miR-629-5p* and *CAV1*, we mutated the 3′UTR sequence of *CAV1* that binds to *miR-629-5p* and constructed luciferase reporter plasmid (*CAV1*-MUT). *miR-629-5p* mimic and the luciferase reporter plasmid containing wild-type or mutated *CAV1* mRNA 3′UTR were co-transfected into Saos2 and HOS cells. Co-transfection of *miR-629-5p* mimic with wild-type *CAV1* mRNA 3′UTR that contained the binding site of *miR-629-5p* showed reduced luciferase activity, while co-transfection of *miR-629-5p* mimic and *CAV1* mutant did not show decreased luciferase activity in either cell line (P<0.001) (Figure 3D). Next, we analyzed the expression of *CAV1* mRNA in the tissue samples collected. Although only a limited number of osteosarcoma samples were analyzed, we found that *CAV1* mRNA was significantly downregulated in the osteosarcoma tissues compared to adjacent healthy tissues (P=0.0009) (Figure 3E). In addition, a negative correlation was identified between *miR-629-5p* and *CAV1* expression in osteosarcoma tissues (P<0.001) (Figure 3F). We also analyzed the expression of *CAV1* mRNA in osteosarcoma cell lines. The results showed that *CAV1* was significantly downregulated in osteosarcoma cell lines (P<0.05) (Figure 3G). These results indicated that *CAV1* may be a potential effector in osteosarcoma and a downstream target of *miR-629-5p*.

### Influence of *miR-629-5p* on osteosarcoma cell lines by targeting *CAV1*

**Figure 4:**
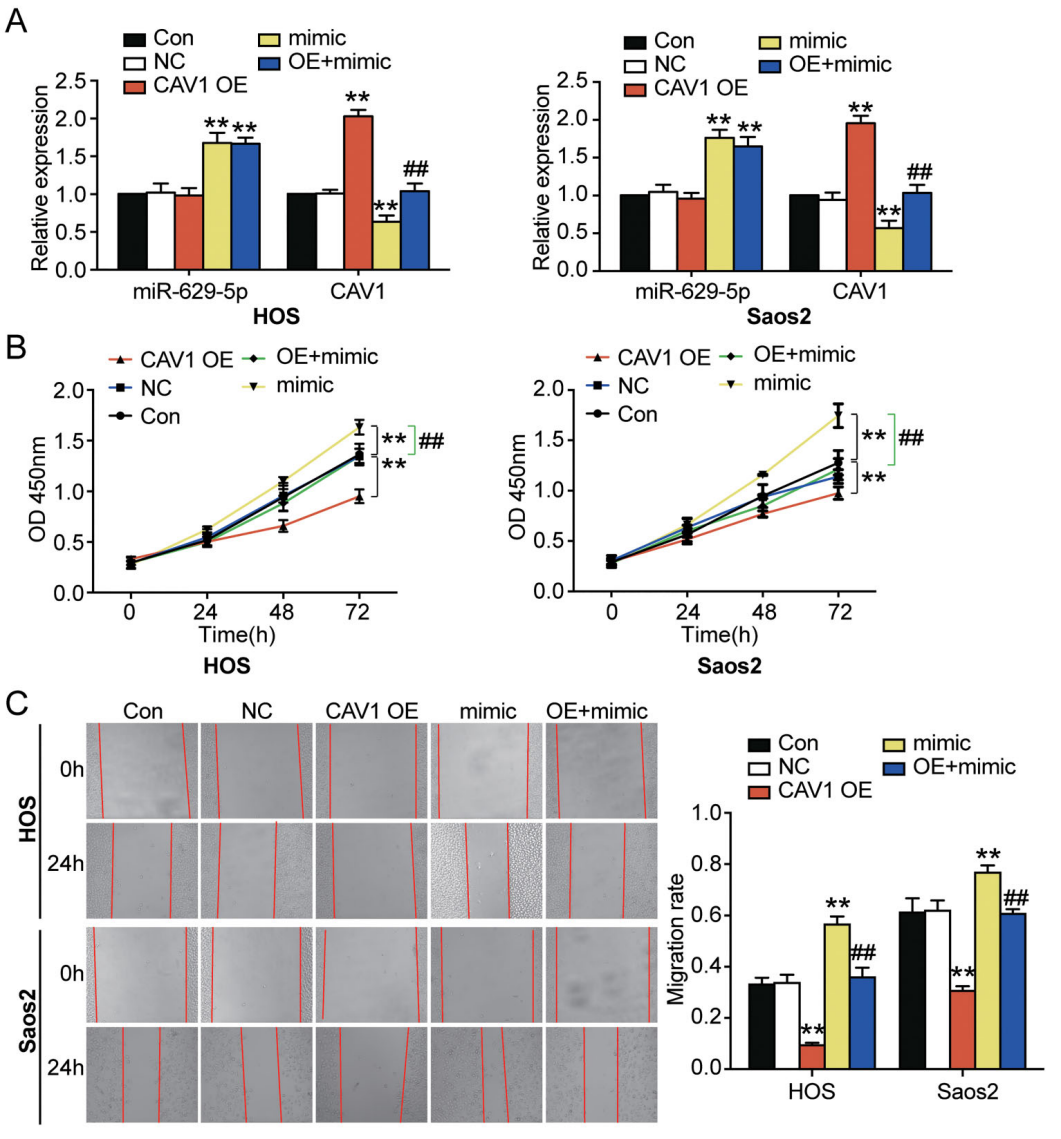
*miR-629-5p* rescued the inhibitory effect of *CAV1* over-expression on osteosarcoma cell phenotypes. **A**, The transfection efficiency of *miR-629-5p* mimic and *CAV1* overexpression plasmids in transfected cells was detected by qRT-PCR. **B**, After transfection with *miR-629-5p* mimic or *CAV1* OE, the proliferation of transfected Saos2 or HOS cells was determined with CCK-8 assay. **C**, The migration rate of the Saos2 or HOS cells transfected with *miR-629-5p* mimic or *CAV1* OE was identified using wound-healing assay. CON: blank control; NC: negative control; mimic: *miR-629-5p* mimic; *CAV1* OE: *CAV1* over-expression. The data of the three independent experiments are reported as means±SD. **P<0.001 compared with the control group.^##^P<0.001 compared with the *CAV1* OE group (ANOVA).

Analyses revealed that *CAV1* OE plasmid transfection increased *CAV1* levels but did not affect *miR-629-5p* expression in either cell line. Transfection of *miR-629-5p* mimic increased *miR-629-5p* but decreased *CAV1* (P<0.001) (Figure 4A). Thus, the qRT-PCR results indicated successful cell transfection. As shown in Figure 4B, *CAV1* upregulation reduced the proliferation of Saos2 and HOS cell lines that was restored by upregulation of *miR-629-5p* (P<0.001). Wound-healing assay demonstrated that *CAV1*-upregulated cells showed impaired migration, but upregulation of *miR-629-5p* significantly restored the reduction in cell migration caused by *CAV1* overexpression (P<0.001) (Figure 4C). These results suggested that *miR-629-5p* promoted the malignant phenotypes in osteosarcoma cell lines by directly inhibiting *CAV1*.

## Discussion

In this study, we demonstrated that *miR-629-5p* and *CAV1* played crucial roles in osteosarcoma development. Specifically, the upregulation of *miR-629-5p* in osteosarcoma tissues and cells promoted osteosarcoma cell proliferation and migration. In addition, *CAV1* was downregulated in osteosarcoma tissues and cell lines, and its overexpression reduced osteosarcoma cell proliferation and migration. Our results showed that an increase in *miR-629-5p* promoted the malignant phenotype of osteosarcoma cell lines by directly suppressing *CAV1*.

*miR-629-5p* upregulation has been found in various cancers (cervical cancer, lymphoblastic leukemia, and colorectal cancer), and it is used as a biomarker for monitoring disease progression (29-31). An abnormal increase in *miR-629-5p* levels stimulates cell proliferation, migration, and invasion and accelerates the aggressiveness of hepatocellular carcinoma cells, thereby resulting in tumor growth and metastasis (18). Interestingly, a study showed that *miR-629-5p* was associated with tipifarnib resistance in breast cancer cells (32). In another study, high levels of *miR-629-5p* in HPV-positive cervical cancer cells were associated with cancer-promoting activities that enhanced E6/E7-dependent cell proliferation and simultaneously reduced apoptosis (33). Furthermore, aberrantly elevated *miR-629-5p* promoted proliferation while inhibiting apoptosis, thus exacerbating and aggravating ovarian cancer (34). In this study, we found that *miR-629-5p* that increased the aggressiveness of osteosarcoma cells was upregulated during the growth of osteosarcoma.

In addition, studies have reported that *miR-629-5p* may potentially promote various oncogenic processes through miRNA-mRNA regulatory networks. In one study, *miR-629-5p* was found to enhance carcinogenesis of human hepatocellular carcinoma by targeting *SFRP2* (18). Another study demonstrated that *miR-629-5p* contributes to ovarian cancer by directly suppressing downstream target genes, including *FGF1*, *AKT3*, or *MAGI2* (35). Moreover, *miR-629-5p* was demonstrated to drive the generation of more aggressive colorectal cancer cells by directly downregulating *CXXC 4*, which affected 5-FU sensitivity (31). Further, the downregulation of *miR-629-5p* inhibits ovarian cancer by targeted inhibition of *TSPYL5*(34). Consistent with these findings, our study found that *miR-629-5p* facilitated the pathological process of osteosarcoma by directly inhibiting the expression of the target gene, *CAV1*. Collectively, these data suggest that *miR-629-5p* may participate in tumorigenesis by downregulating the expression of certain molecules.

A previous study found that abnormal expression of *CAV1* is associated with the degree of malignancy of osteosarcoma cells, with *CAV1* downregulation in highly malignant cells (36). In this study, we found that *CAV1* was downregulated in osteosarcoma tissues. To date, inactivation of *CAV1* has been shown to be associated with the occurrence of multiple types of malignancies (37). In addition, *CAV1* was shown to reduce paclitaxel resistance in osteosarcoma cells by attenuating PI3K-Akt-JNK-dependent autophagy (38). In this study, we found that *CAV1* overexpression repressed the malignant phenotype in osteosarcoma cells. Our results were similar to a study that showed that *CAV1* inhibited osteosarcoma cell (Saos-2 cell line) proliferation and invasion by upregulating the calcium sensing-receptor CaSR (39). Another study found that *CAV1* that suppresses the progression of ovarian cancer is directly inhibited by miR-96-5p, indicating that *CAV1* may be regulated by miRNAs in carcinogenesis (40).

This study had some limitations. For instance, our study did not explore the downstream regulatory network involving *miR-629-5p* and *CAV1*. Animal experiments have not yet been conducted to verify the malignant behavior of tumors. Moreover, clinical analysis is needed to determine whether *miR-629-5p* has the potential to aggravate tumor metastasis. Nonetheless, our results confirmed that *miR-629-5p* stimulates the malignant phenotype of osteosarcoma cell lines by directly downregulating *CAV1*. In addition, our findings suggested that the *miR-629-5p*-*CAV1* interactome represents a potential new target for osteosarcoma treatment.

## Supplementary Material

Click here to view [pdf].
